# A brain-inspired object-based attention network for multiobject recognition and visual reasoning

**DOI:** 10.1167/jov.23.5.16

**Published:** 2023-05-22

**Authors:** Hossein Adeli, Seoyoung Ahn, Gregory J. Zelinsky

**Affiliations:** 1 Department of Psychology, Stony Brook University, Stony Brook, NY, USA; 2 Department of Computer Science, Stony Brook University, Stony Brook, NY, USA

**Keywords:** object-based attention, object-centric representation, object recognition, deep neural networks, visual pathways, visual reasoning

## Abstract

The visual system uses sequences of selective glimpses to objects to support goal-directed behavior, but how is this attention control learned? Here we present an encoder–decoder model inspired by the interacting bottom-up and top-down visual pathways making up the recognition-attention system in the brain. At every iteration, a new glimpse is taken from the image and is processed through the “what” encoder, a hierarchy of feedforward, recurrent, and capsule layers, to obtain an object-centric (object-file) representation. This representation feeds to the “where” decoder, where the evolving recurrent representation provides top-down attentional modulation to plan subsequent glimpses and impact routing in the encoder. We demonstrate how the attention mechanism significantly improves the accuracy of classifying highly overlapping digits. In a visual reasoning task requiring comparison of two objects, our model achieves near-perfect accuracy and significantly outperforms larger models in generalizing to unseen stimuli. Our work demonstrates the benefits of object-based attention mechanisms taking sequential glimpses of objects.

## Introduction

Objects are the units through which we interact with the world and perform tasks (Spelke, 1990; Peters & Kriegeskorte, 2021; Scholl, 2001). Consider the simple task of searching for an object in a cluttered environment (e.g., a paper clip in a busy drawer). This task requires one to sequentially move spatial attention to select objects and to use object-based attention to route and bind the features of these objects so that they can be recognized as a target or nontarget. The role of the attention mechanism in this process is to make the objects in the sequence the figure, in order to facilitate interacting with the object and performing the task (Scholl & Pylyshyn, 1999; Vecera, 2000). Works have also suggested that object-based attention is important for achieving human-level performance in accuracy and generalization (George et al., 2017; Scholl, 2001). However, in contrast to spatial (Desimone & Duncan, 1995; Adeli, Vitu, & Zelinsky, 2017) or feature-based attention (Maunsell & Treue, 2006; Lindsay & Miller, 2018; Adeli & Zelinsky, 2018), where attention studies can focus on simple, well-defined visual features (e.g., location, color, etc.), the units of selection for object-based attention (Baldauf & Desimone, 2014; Ekman, Roelfsema, & de Lange, 2020; Lei, Benjamin, & Kording, 2021) are entities consisting of complex spatial properties (shapes) and distributed feature representations. Moreover, perception is akin to hypothesis testing, where a top-down modulation reflecting object hypotheses and goals affects object selection and bottom-up processing. Leveraging recent developments in deep learning (LeCun, Bengio, & Hinton, 2015), here we model a general-purpose object-based attention mechanism, requiring solutions to three subtasks: (a) solving the binding problem, grouping features of each object, and using object-centric representation learning; (b) capturing the interaction between a (largely) bottom-up mechanism for recognition and a top-down mechanism for attention planning using encoder–decoder models; and (c) learning to sequentially sample objects using end-to-end training.

To solve the object binding problem (Treisman, 1996), recent deep neural networks (DNNs) have been proposed that create object-centric representations of the entities in a scene by spatially segregating the features from the background and dynamically grouping those features in spatial and representational domains (Greff, van Steenkiste, & Schmidhuber, 2020). This segregation and grouping relies on (bottom-up) part-whole matching and Gestalt processes interacting with (top-down) objectness priors and knowledge of object categories (Greff et al., 2020; Vecera, 2000; Wagemans et al., 2012). Objects are represented in separate “slots” (Greff et al., 2020; Goyal et al., 2019; Burgess et al., 2019; Locatello et al., 2020; Eslami et al., 2016), realizing the cognitive concept of “object files” (Kahneman, Treisman, & Gibbs, 1992; Goyal et al., 2020). A recent development in this domain, capsule networks (CapsNets) (Sabour, Frosst, & Hinton, 2017; Hinton, Sabour, & Frosst, 2018), attempts to represent scenes as parse trees. Capsules in different layers represent visual entities at different levels of object granularity in the image, from small object parts in the lower levels to whole objects at the top level. Capsules provide an encapsulation and grouping of object representations that has shown benefits for performing object-based and class-conditioned reasoning for downstream tasks (e.g., deflecting adversarial attacks; Qin, Frosst, Raffel, Cottrell, & Hinton, 2020). In this context, we adopt the following definitions. We refer to “binding” as the encapsulation of visual feature information in the slot for a given object, meaning that the object is responsible for the information in the slot becoming bound into a representation. By “object representation,” we refer to the totality of bound information in an object’s slot, and we evaluate a learned object representation by using it to reconstruct the object. These formulations do not capture certain aspects of bound object representations (e.g., a three-dimensional structure), yet they still have broad implications for the modeling of visual perception (e.g., the prediction of global shape perception by humans; Doerig, Schmittwilken, Sayim, Manassi, & Herzog, 2020; Rodríguez-Sánchez & Dick, 2019).

An object-centric perspective requires an integrated attention-recognition mechanism that is nicely embodied by the interacting structures organized along the ventral and dorsal visual pathways in the brain (Ungerleider & Haxby, 1994; Ungerleider & Pessoa, 2008). The ventral “what” pathway is involved in feature processing and recognizing objects and scenes (Felleman & Van Essen, 1991; Ungerleider & Haxby, 1994). Core object recognition refers to the rapid recognition of briefly presented objects (100 ms) (VanRullen & Thorpe, 2001) and is believed to be carried out primarily in the initial feedforward processing pass in the ventral pathway (DiCarlo, Zoccolan, & Rust, 2012). Modeling works support this claim by showing that neural activation along this pathway during core object recognition can be predicted (Cadieu et al., 2014; Güçlü & van Gerven, 2015; Cichy, Khosla, Pantazis, Torralba, & Oliva, 2016) using feedforward convolutional neurals networks (CNNs; LeCun, Bottou, Bengio, & Haffner, 1998; LeCun et al., 2015) trained on the same recognition tasks. However, difficult recognition tasks require recurrent and feedback connections beyond the feedforward pass (Kietzmann et al., 2019; Kar & DiCarlo, 2021; Spoerer, McClure, & Kriegeskorte, 2017; Wyatte, Jilk, & O’Reilly, 2014). The dorsal “where” pathway is believed to be involved in the spatial prioritization of visual inputs and the guidance of actions to objects (Bisley & Goldberg, 2010; Szczepanski, Pinsk, Douglas, Kastner, & Saalmann, 2013). Here we take inspiration from the role played by dorsal structures in providing attentional modulation of ventral pathway activity, as shown in previous work (Deco & Rolls, 2004; Bisley & Goldberg, 2010). The attention signal generated in the dorsal pathway prioritizes and modulates the routing of the visual inputs in the ventral pathway for the purpose of improving object classification success and better performing the task. The repetition of this process imposes seriality on behavior when confident classification decisions are needed, making visual perception a sequential process. Previous neurocognitive modeling work on an integrated attention-recognition mechanism (Deco & Rolls, 2004) has been largely hand designed and applied only to simpler stimuli, limitations that could be better addressed with a learning-based approach.

Our model of object-based attention employs the general structure of autoencoders (and more generally encoder–decoders). This class of models learns to encode the sensory input into a compact representation that captures the important aspects of the input and then decode that representation to reconstruct the sensory input (or translate it into another modality, e.g., image to text) (Kingma & Welling, 2013; Xu et al., 2015). Notably, the DRAW  (Gregor, Danihelka, Graves, Rezende, & Wierstra, 2015) architecture introduced a sequential spatial attention mechanism to variational autoencoders (VAEs; Kingma & Welling, 2013) and showed that the model can learn to iteratively glimpse different parts of the input images and reconstruct them. Our premise is that the encoder–decoder framework loosely maps onto interactions between the dorsal and ventral processing in the human brain’s attention-recognition system. We believe core object recognition along the ventral pathway can be mapped to encoder processing. Decoder processing maps onto dorsal pathway, from which originates the top-down attention signal that modulates ventral activity. These encoder and decoder steps are taken iteratively, creating a repeating cycle of prioritization and selection. Within this framework, we present OCRA, an object-centric recurrent attention model that combines recurrent glimpse-based attention and object-centric representation learning. Like a CapsNet, it performs encapsulation of features to structure the higher-level representations for object processing. However, we place this structure within the aforementioned encoder–decoder model with recurrent attention, thereby enabling integration of structured information across multiple attentional glimpses. We show that capsule-based binding of object features and grouping is effective in the sequential detection of multiple objects. It is also very effective in performing visual reasoning tasks (judging whether two randomly generated shapes are the same or different; Fleuret et al., 2011) and on a challenging generalization task where the model is tested on stimuli that are different from the training set.

## Materials and methods

### OCRA architecture

**Figure 1.**
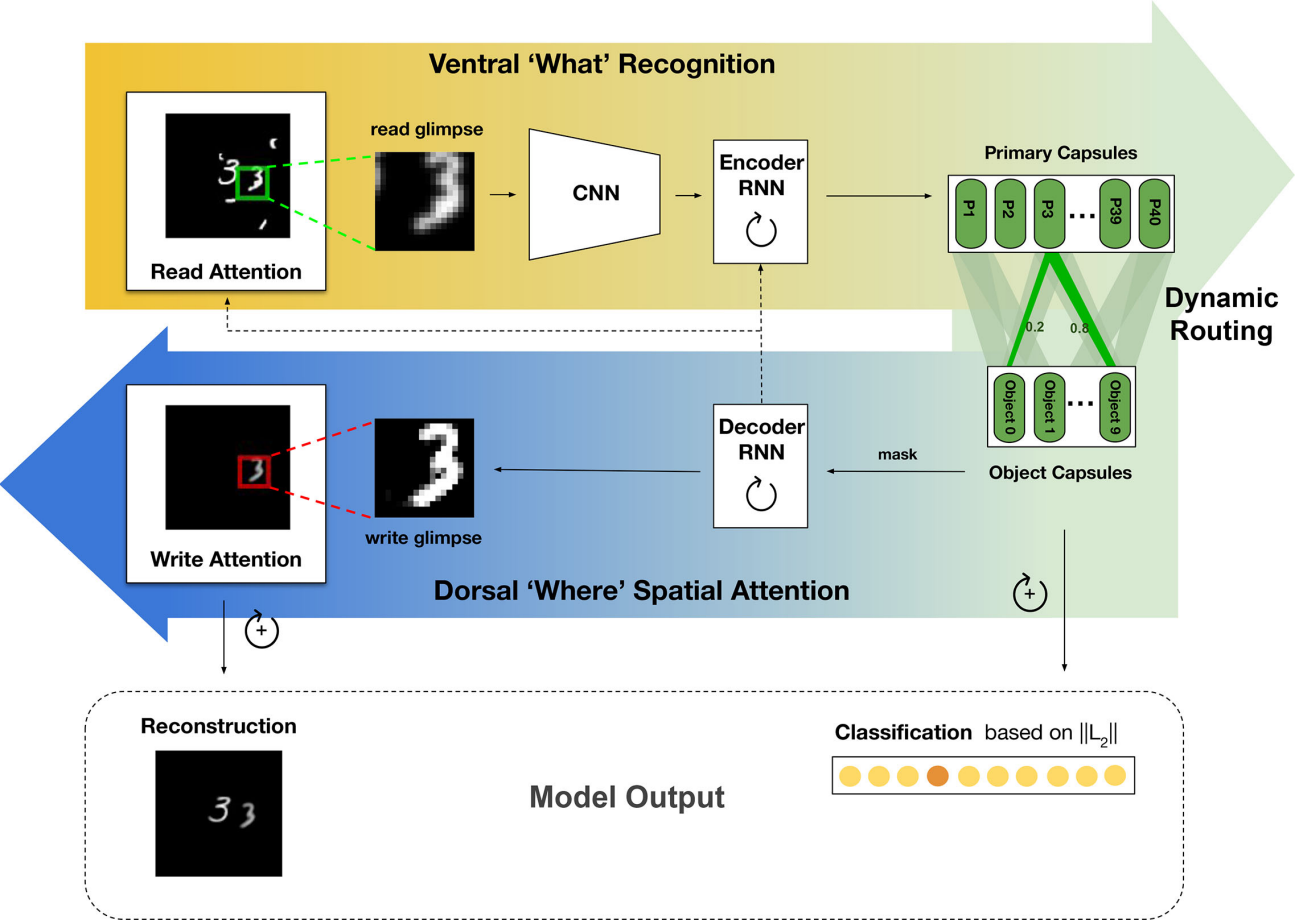
OCRA architecture. At each timestep of ventral processing, the encoder inputs a new read glimpse that is taken from the image using an attention window specified by the decoder RNN. The read glimpse is first processed using a small CNN network to obtain features that are then input to a recurrent layer. The primary capsules are read from this recurrent activation using a linear mapping and are dynamically routed through agreement to the class capsules. The magnitude of the class capsules determines the evidence for a class in the selected glimpse. For the dorsal processing, the class (digit) capsules are then masked to only route the most active capsule to the decoder RNN where the representations are maintained over timesteps. This representation is used to plan attentional glimpses for the reading operation–what gets routed in the ventral pathway and also using a similar mechanism for reconstructing the image by generating where and what to write to the canvas. The connection from decoder RNN to encoder RNN allows the ongoing recurrent representation to also further modulate routing in the ventral pathway. We only use this connection for the visual reasoning task. This sequential process is iterated for multiple steps, after which the cumulative magnitude of the class capsules determines the final classification. Both the class capsules and the cumulative canvas are used for training.

**Figure 2.**
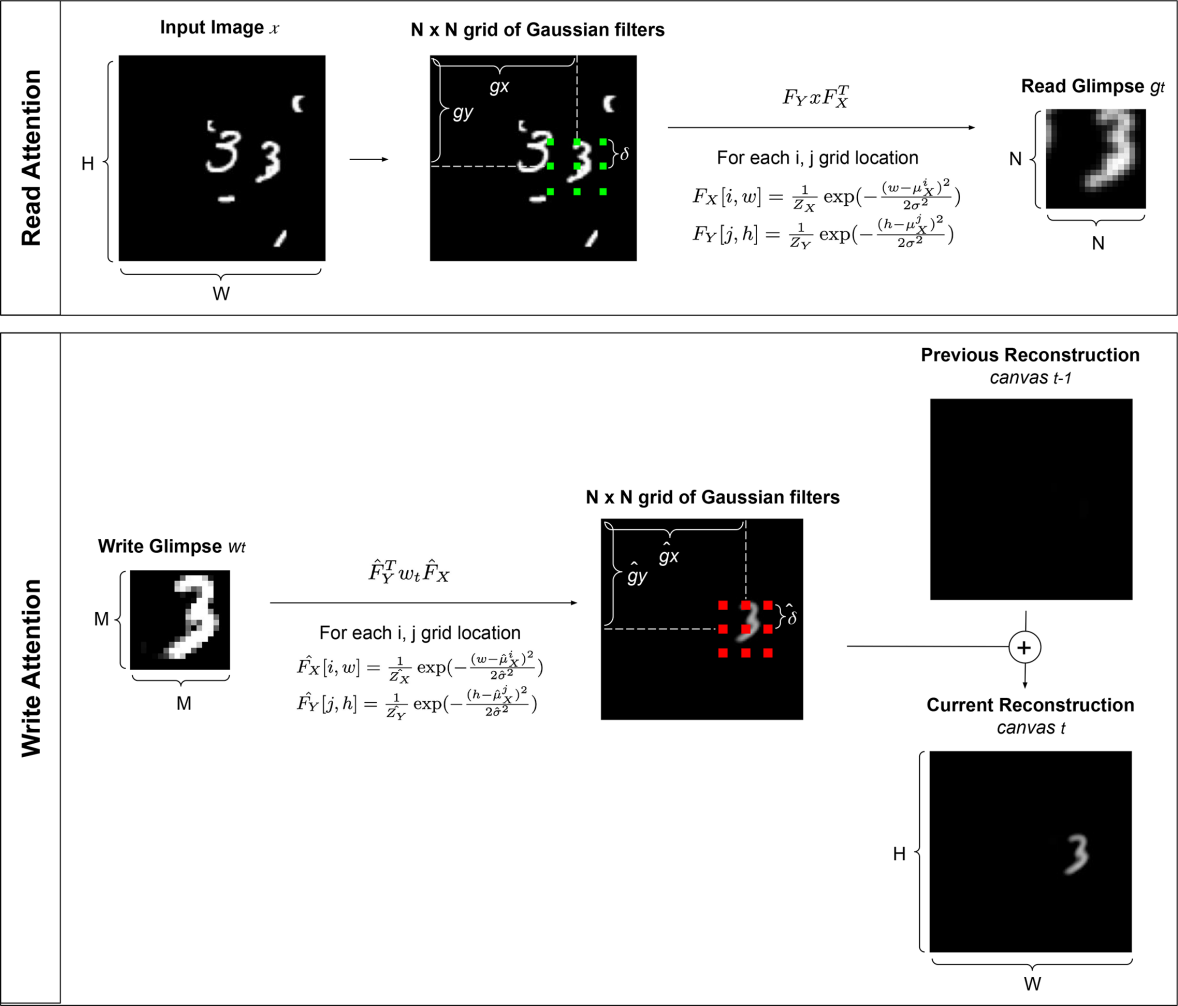
Visual illustrations of read and write attention mechanisms.

The architecture for OCRA is shown in Figure 1. Building on the DRAW model (Gregor et al., 2015), the “attention window” in OCRA is a grid of filters applied to a variable-sized area of the image. However, because the number of filters covering the attention window remains constant, as the window gets bigger, it samples increasingly low-resolution information, creating a trade-off between the size of the attention window and the resolution of information extracted. This property is aligned with “zoom lens” theories of human attention (Eriksen & James, 1986; Müller, Bartelt, Donner, Villringer, & Brandt, 2003) that similarly propose a trade-off between resolution and a variable-sized attention process that can be broadly or narrowly allocated to an input (see Figure 2 for a visual illustration of the attention mechanism). The original DRAW model (Gregor et al., 2015) was formulated as an autoregressive VAE, as it was trained for stepwise self-supervised reconstruction. In contrast, our formulation uses a deterministic encoder–decoder approach. We train OCRA to predict both the category classification, based on object-centric capsules, and an image reconstruction, based on decoder output. An overview of the OCRA components, loss functions, and implementation details is provided in this section. A pytorch (Paszke et al., 2019) implementation of OCRA with additional details and results is provided.^1^

####  

##### Read and write attention

At each timestep, a glimpse, *g*_*t*_, is sampled through applying a grid of N × N Gaussian filters on the input image *x*. We set the glimpse size to 18 × 18 for our experiments, with a sample glimpse shown in Figure 1 (left). The Gaussian filters are generated using four parameters: *g*_*X*_, *g*_*Y*_, δ, σ^2^, which specify the center coordinates of the attention window, the distance between equally spaced Gaussian filters in the grid, and the variance of the filters, respectively. All of these parameters are computed via a linear transformation of the previous step decoder RNN (recurrent neural network) output $h_{t-1}^{dec}$ using a weight matrix *W*_*read*_, which makes the attention mechanism fully differentiable. A similar procedure applies to the *write* attention operation. The decoder RNN output $h_{t}^{dec}$ is linearly transformed into an M × M write patch *w*_*t*_ (set to 18 × 18 in our experiments), which is then multiplied by the Gaussian filters to reconstruct the written parts in the original image size (Figure 1, right). The Gaussian filters used for the *write* operation differed from those used for the *read* operation and were computed from four parameters obtained from a separate linear transformation, $W_{write\_attention}$, of the decoder RNN output $h_{t}^{dec}$. An illustration of the read and write attention operations is provided in Figure 2.

##### Encoder

After a glimpse is selected from the input image by the read attention operation, it is processed first using a two-layer CNN with 32 filters in each layer. Kernel sizes were set to 5 and 3, respectively, for the first and the second layers. Each convolutional layer is followed by max pooling with a kernel size of 2 and a stride of 2, and rectified linear units (ReLU) were used for nonlinear activation functions. Given the glimpse size of 18 × 18, the resulting 32 feature maps are of size 4 × 4. The feature maps, $g_{t}^{conv}$, are reshaped (to a vector of size 512) and used as input to the encoder RNN, along with the encoder RNN hidden state from the previous step, $h_{t-1}^{enc}$. We used long short-term memory (LSTM) (Hochreiter & Schmidhuber, 1997) units (size 512) for the recurrent layers in our model.

##### Latent capsule representations and dynamic routing

We use a vector implementation of capsules (Sabour et al., 2017) where the magnitude of the vector represents the existence of the visual entity and the orientation characterizes its visual properties. The primary-level capsules are generated through a linear readout of the encoder RNN, $h_{t}^{enc}$. These capsules are meant to represent lower-level visual entities (“parts”) that belong to one of the higher-level capsules in the object capsule layer (“whole”). To find this part–whole relationship, we used the dynamic routing algorithm proposed by Sabour et al. (2017). Dynamic routing is an iterative process where the assignments of parts to whole objects (coupling coefficients) are progressively determined by agreement between the two capsules (measured by the dot product between the two vector representations). Each primary level capsule (*i*) provides a prediction for each object-level capsule (*j*). These predictions are then combined using the coupling coefficients (*cij*) to compute the object-level capsule. Then the agreement (dot product) between the object-level capsules and the predictions from each primary-level capsule impacts the coupling coefficients for the next routing step. For example, if the prediction for a digit capsule *j* from a primary capsule *i*, (${\hat{p}}_{t}^{j|i}\leftarrow W_{t}^{ij}p_{t}^{i}$), highly agrees with the computed digit capsule $\left(\sum_{i}c_{t}^{ij}{\hat{p}}_{t}^{j|i}\right)$, the coupling coefficient $c_{t}^{ij}$ is enhanced so that more information is routed from primary capsule *i* to object capsule *j*. Coupling coefficients are normalized across the class capsule dimension following the max–min normalization (Zhao, Kleinhans, Sandhu, Patel, & Unnikrishnan, 2019) as in Equation 1. Lower and upper bounds for normalization, *lb* and *ub*, were set to 0.01 and 1.0. This routing procedure iterates three times. We used this method instead of the softmax normalization in Sabour et al. (2017) because we observed the latter method would not differentiate between the coupling coefficients. In our experiments, we used 40 primary-level capsules, each a vector of size 8. The object capsules are vectors of size 16, and there are 10 of them corresponding to the 10-digit categories for the multiobject recognition task and 4 of them for the visual reasoning task. For the object-level capsules, we use a squash function (Equation 2) to ensure that its vector magnitude is within the range of 0 to 1. For the mutliobject recognition task, these would represent the probability of a digit being present in the glimpse at each step. Once the routing is completed, we compute the vector magnitude (L2 norm) of each object capsule to obtain classification scores. The final digit classification is predicted based on the scores accumulated over all timesteps. For the visual reasoning task, two capsules (among four total) were designated to be the response capsules, and the cumulative magnitude of these capsules was used for predicting the same–different responses.

(1) $$c_{t}^{ij}=lb+\left(ub-lb\right)\frac{c_{t}^{ij}-min\left(c_{t}^{ij}\right)}{max\left(c_{t}^{ij}\right)-min\left(c_{t}^{ij}\right)}$$

(2) $$d_{t}^{j}=\frac{∥v_{t}^{j}{∥}^{2}}{1+∥v_{t}^{j}{∥}^{2}}\frac{v_{t}^{j}}{∥v_{t}^{j}∥}$$

##### Decoder

The object capsules provide a structured representation that can be used for decoding and glimpse selection. We first mask the object capsules so that only the vector representation from the most active capsule is forwarded to the decoder RNN, which also inputs the hidden state from the previous step, $h_{t-1}^{dec}$. Because the decoder maintains through recurrence the ongoing and evolving object-based representation of the image, it is best suited to determine the next read glimpse location (as discussed earlier). The state of the decoder RNN is also used through two linear operations to determine what and where to write in the canvas to reconstruct the image.

### Loss function

OCRA outputs object classification scores (cumulative capsule magnitudes) and image reconstruction (cumulative write canvas). Losses are computed for each output and combined with a weighting as in Equation 3. For reconstruction loss, we simply computed the mean squared differences in pixel intensities between the input image and the model’s reconstruction. For classification, we used margin loss (Equation 4). For each class capsule *j*, the first term is only active if the target object is present (*T*_*j*_ > 0) where minimizing the loss pushes the capsule magnitude to be bigger than target capsule magnitude minus a small margin (*m*). The second term is only active when the target capsule magnitude is zero, and in that case, minimizing the loss pushes the predicted capsule magnitude to be below a small margin (*m*). For all the experiments in this article, we used an Adam optimizer (Kingma & Ba, 2014).

(3) $$\begin{array}{c} \;\;\;\;\;\;\;\;\;\;\;\;\;Total\;Loss=\sum_{j\in class}Class\;Loss_{j}+\lambda_{recon}Recon\;Loss\; \end{array}$$

(4) $$\begin{array}{ccc} \;\;\;\;\;\;\;\;\;\;\;\;\;Class\;Loss & \;= & \sum_{j\in class}max\left(0,min\left(T_{j},1\right)\right)\cdot max(0,\left(T_{j}-m\right)\;\\ & & -∥d_{j}{∥)}^{2}+\lambda_{absent}\cdot max\left(0,1-T_{j}\right)\\ & & \cdot max(0,∥d_{j}{∥-m)}^{2} \end{array}$$

### Stimuli generation

####  

##### MultiMNIST-cluttered dataset

We generated the MultiMNIST-cluttered dataset to be similar to the cluttered translated MNIST dataset from Mnih, Heess, Graves, and Kavukcuoglu (2014). For each image, two digits and six digit-like clutter pieces are placed in random locations on a 100 × 100 blank canvas. All digits were sampled from the original MNIST dataset (LeCun et al., 1998), and the two digits in each image could be from the same or different categories. Clutter pieces were generated from other MNIST images by randomly cropping 8 × 8 patches. We generated 180K images for training and 30K for testing, ensuring to maintain the same MNIST training/testing separation.

##### MultiMNIST dataset

We generated the MultiMNIST dataset following the method from Sabour et al. (2017). Each image in this dataset contains two overlapping digits sampled from different classes of the MNIST handwritten digits dataset (LeCun et al., 1998) (size 28 × 28 pixels). After the two digits are overlaid, each is shifted randomly up to 4 pixels in horizontal and vertical directions, resulting in images of size 36 × 36 pixels with on average 80% overlap between the digit bounding boxes. We generated 3 million images for training and 500K images for testing and ensured that the training/testing sets were generated from the corresponding MNIST sets (i.e., the training set in MultiMNIST was only generated from the training set in MNIST).

##### SVRT Task 1

We generated the Synthetic Visual Reasoning Test (SVRT) stimuli using the code from Fleuret et al. (2011). We generated 60K images for training and validation and 10K images for testing. The images are 64 × 64 pixels. The stimuli for the generalization task were generated using the code modified from Puebla and Bowers (2022). For each of the nine types, we generated 6K images for training (54K in total) and 1.2K for testing (10.8K in total). We also generated 5.4K images for the four out-of-distribution (OOD) shape types that were only used for testing.

## Results

Object-based attention mechanisms endow a model with the ability to effectively segregate and represent different objects in order to perform a task. To test this in OCRA, we used multiobject recognition and visual reasoning tasks. First, the model’s ability to segregate the figure objects from background clutter is tested on the MultiMNIST-cluttered dataset (Ba, Mnih, & Kavukcuoglu, 2015). Second, we test the model’s recognition performance when objects are highly overlapped (and thus occluded) using the MultiMNIST task (Sabour et al., 2017). We also examine the model’s performance on learning visual reasoning using a paradigm similar to Task 1 of SVRT (Fleuret et al., 2011), where the model must detect whether two randomly generated objects in a given scene are the same or different. All model accuracy results reported in this section are averages of five runs (see supplementary material for training details and hyperparameter selection for different experiments). For recognition tasks, accuracy is measured on the image level, meaning that the response is correct only if both objects in an image are recognized correctly.

### Objects in clutter

Recognizing an object in a noisy environment requires the grouping and binding of the object’s features and their segregation from the noisy background and potentially other objects. We tested whether our model could learn to bind and segregate objects using the MultiMNIST-cluttered task. The stimuli for this task are generated by placing two random digits and multiple object-like distractor pieces on a canvas (see Stimuli generation).

**Figure 3.**
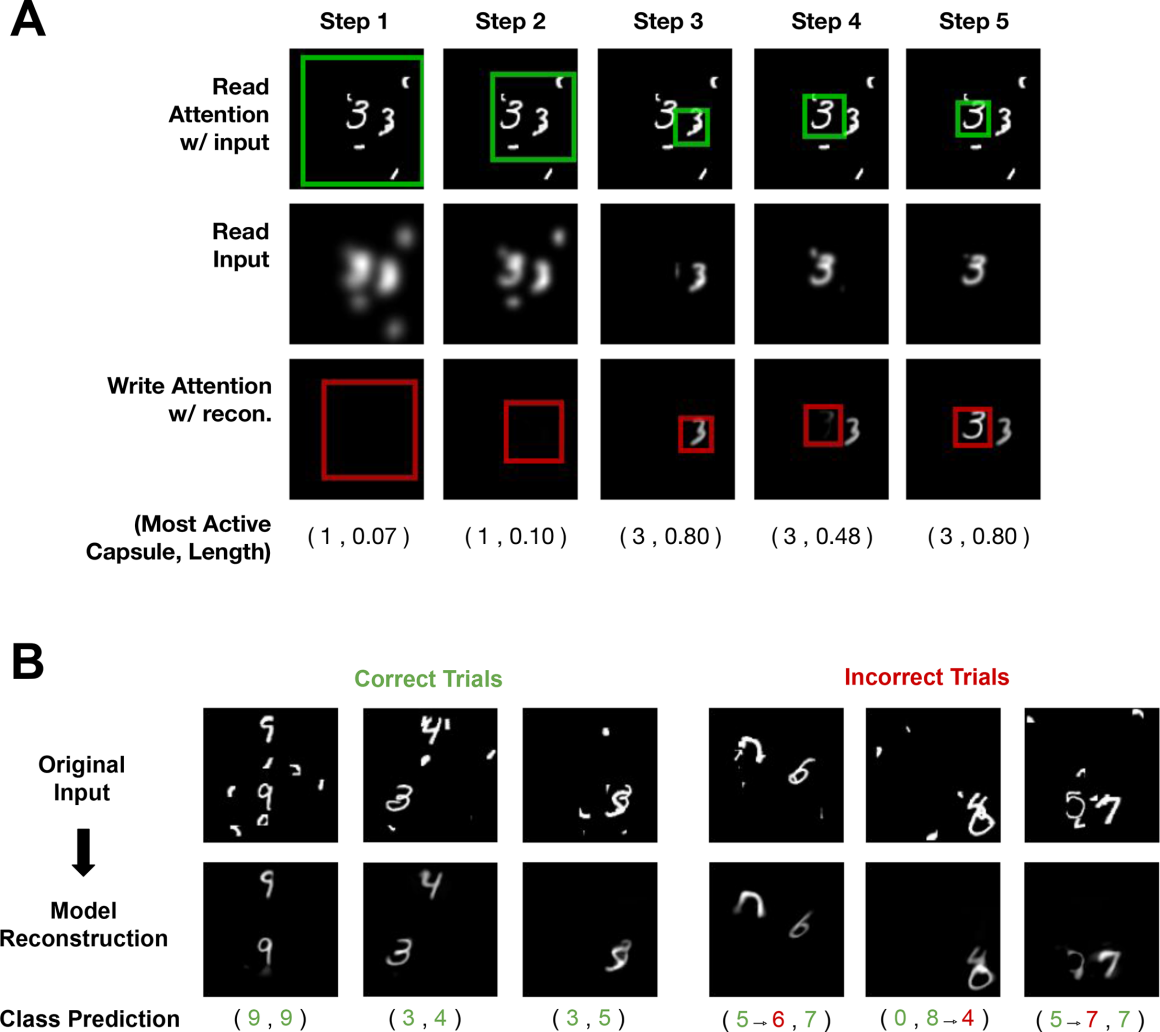
(**A**) Stepwise process of moving the glimpse over a sample MultiMNIST-cluttered image. The read attention windows are overlaid as green boxes on the original image in the first row. The read glimpse is shown in the second row, and the bottom row shows the cumulative canvas and the location of write attention as a red box. The most active capsule and the corresponding capsule magnitudes are displayed at the bottom for each step. (**B**) Sample classification and reconstruction predictions for OCRA on the MultiMNIST-cluttered task. The green digits show the ground truth and correct model predictions, and erroneous predictions from the model are in red.

Figure 3A shows OCRA’s ability to recognize the two digits over five timesteps for a sample image from this task. The top row shows the size and location of the attention window for each step. Note that OCRA makes its initial glimpse (left column) large (and therefore spatially biased to the center) so as to cover most of the image, presumably to obtain its version of scene gist. The second row shows the information being read from each attention glimpse, in image dimensions. The blurring observed over Steps 1 to 3 illustrates the aforementioned zoom–lens relationship between the size of the glimpse window and its resolution, owed to OCRA’s attention having a constant number of filters. We define a reconstruction mask that is the averaged sum of all the read glimpses converted into image dimensions (average of Figure 3A, middle row). This mask would specify the image areas that the model had glimpsed by the end of each trial and is used during training to only penalize the model for not reconstructing those areas. In other words, this mask effectively focuses the loss so that the model is accountable for reconstructing only the areas where it had glimpsed, allowing the model to be selective with its glimpses and write operations and avoid glimpsing at distractors. The most active capsule that is routed to the decoder at each step, and its magnitude, is provided at the bottom.

OCRA learns to take sequential glimpses of the image at different scales to detect and classify the digits. Additionally, it learns to write these glimpses to the canvas only when it is confident of the digit classification. As Figure 3A shows, this interacting attention-recognition process enables OCRA to recognize digits embedded in considerable noise. Note also that the model learns that recognition requires the sequential disengagement of attention from one object and the movement of the glimpse window to the other object so that it too can be recognized, as illustrated by the object-centric additions to the canvas in the figure. Note that this serial behavior parallels the fact that human object recognition is also serial, at least for objects complex enough to require a feature binding (Treisman, 1996). Figure 3B shows more examples of model predictions, with correct responses on the left side and errors on the right. Most errors by OCRA on this task are due to the digits overlapping with each other or with noise pieces in ways that change their appearance from the underlying ground truth. Supplementary Figure S1 shows the glimpse-based reconstruction for few sample images with correct model predictions.

OCRA performed the MultiMNIST-cluttered task with 94% accuracy, which is comparable to other glimpse-based models (Mnih et al., 2014; Ba et al., 2015). While this broadly confirms the value of including a glimpse-based attention mechanism, there are notable differences in our approach. First, OCRA’s attention mechanism is differentiable and therefore easy to train, in contrast to the reinforcement learning-based mechanisms employed in earlier work. Another difference is the use of a context network in Ba et al. (2015) that inputs the whole image to the model in a separate pathway to plan attention selection, thereby separating it from the recognition pathway. OCRA has one pathway and strings together glimpses from multiple steps to have an integrated recognition and attention planning mechanism. Our model does this through its “zoom lens” attention processes that can switch between local and global scales as needed. OCRA’s early glimpses are taken from the whole image, providing the model with a low-resolution gist description (Figure 3A, middle row, left) that allows it to plan future glimpses. Reflecting its object-centric design, OCRA’s later glimpses are focused on individual objects to mediate the recognition and reconstruction processes.

### Overlapping objects

**Figure 4.**
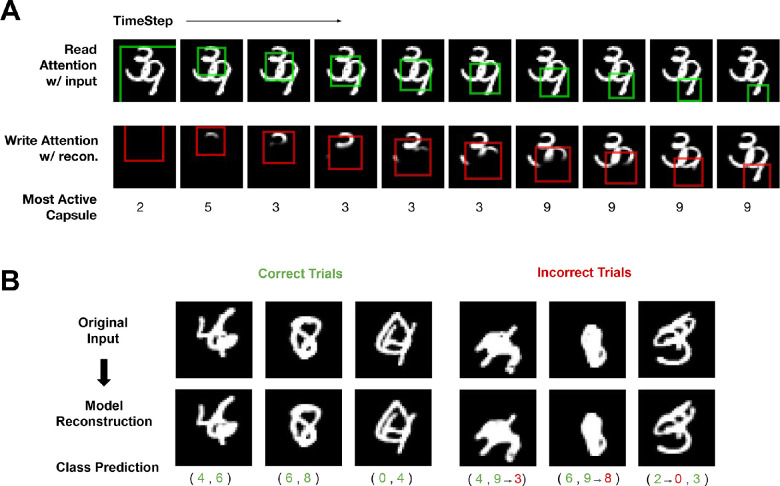
(**A**) Stepwise process of moving the glimpse over a sample MultiMNIST image. The read attention windows are overlaid as green boxes on the original image in the first row. The second row shows the cumulative canvas and the location of write attention as a red box with the most active capsule for each step displayed at the bottom. (**B**) Sample classification and reconstruction predictions for OCRA on the MultiMNIST task. The green digits show the ground truth and correct model predictions and erroneous predictions from the model are in red.

Objects commonly occlude each other, and to study the attention-recognition system under these conditions, we used the MultiMNIST task. This task involves recognizing digits having an average of 80% overlap between their bounding boxes. Figure 4A shows OCRA’s glimpse behavior on a sample image. The model starts with a more global glimpse but then moves its attention window, first to one object and then to the other, recognizing and reconstructing each sequentially. The most active capsule at each processing timestep is indicated by the digits below the second row. The gradual spreading of the reconstruction shown in the bottom row is consistent with the spreading of attention within an object, a pattern hypothesized by object-based models of attention (Jeurissen, Self, & Roelfsema, 2016; Ekman et al., 2020). However, the gradual reconstruction does not show a clean cut between the two objects in the cumulative canvas, which we believe is due to the model having only seen the objects in these highly occluded images in this task (and never in isolation) and not being provided with object segmentations (only the classification ground truth). Figure 4B shows samples of the model making correct and incorrect predictions, as well as the resulting reconstructions. Table 1 shows OCRA’s overall accuracy in this task (whether both objects in the image are correctly classified) compared to the CapsNet model (Sabour et al., 2017) where the MultiMNIST task was originally introduced. Our model with three glimpses significantly outperforms the CapsNet model while having only one third the number of parameters. Finally, we observed a clear effect of the number of timesteps on model performance, with the error rate dropping from 3 timesteps to 10 timesteps. Just as human recognition benefits from longer attention sampling when discriminations are difficult, so too does OCRA’s performance.

**Table 1.** MultiMNIST classification accuracy.

Model	Model size (# parameters)	Average accuracy (*SEM*)
CapsNet (Sabour et al., 2018)	11.36M	87.3% (±0.16)
OCRA-3glimpse	3.87M	92.76% (±0.11)
OCRA-10glimpse	3.87M	94.92% (±0.17)

To more clearly understand the model dynamics, we conducted experiments starting from the OCRA-3glimpse model and ablated different model components. Namely, we conducted separate ablations for the object-centric representation, the sequential glimpse mechanism, and the model’s recurrent processing, to measure their impact on model performance. Results are shown in Table 2.

**Table 2.** Ablation study results on MultiMNIST classification.

Model	# of glimpses	Capsules	Model size	Average accuracy (*SEM*)
OCRA (proposed)	3	Yes	3.87M	92.76% (±0.11)
OCRA-Recurrent	No	Yes	8.58M	91.02% (±0.14)
OCRA-Feedforward	No	Yes	6.47M	89.37% (±0.10)
OCRA-no Capsules	3	No	3.87M	91.96% (±0.13)

####  

##### The role of recurrence and attention glimpse mechanisms

In the first ablation experiment, we asked how OCRA’s performance compares to a recurrent model that uses object-centric representation but lacks an ability to obtain glimpse samples. OCRA-Recurrent (Table 2) performs multistep processing on the input image using the recurrence in its encoder and decoder RNNs but cannot glimpse at specific locations. The model therefore receives the entire image as input at each processing step, which also requires it to have more parameters (36 × 36 pixel input compared to 18 × 18 pixel glimpse). We then trained this model using three timesteps to make it comparable to OCRA-3glimpse. As shown in Table 2, accuracy for OCRA-Recurrent is lower than for OCRA-3glimpse, highlighting the importance of the glimpse mechanism in our model performance. We next removed the recurrence mechanism completely from OCRA, leaving a model that makes one feedforward pass with the full-resolution image as its input. This feedforward model then binds features for each object in separate category capsules before feeding them to the decoder (without masking the object capsules) to reconstruct the whole image at once. Table 2 shows that this feedforward model is not only less accurate than OCRA but also less accurate than OCRA-Recurrent. This comparison broadly supports previous work arguing for a role of recurrent dynamics in assisting recognition tasks involving high degrees of occlusion (Spoerer et al., 2017; Wyatte et al., 2014). This benefit of recurrence on object recognition is hypothesized to be due to recurrence, providing more computational depth (Nayebi et al., 2022; van Bergen & Kriegeskorte, 2020; Schwarzschild, Gupta, Ghiasi, Goldblum, & Goldstein, 2021) and leveraging contextualized iterative computations (van Bergen & Kriegeskorte, 2020). Taken together, while our results show that recurrent computation can improve accuracy in challenging recognition tasks with high occlusion, pairing recurrence with an attention glimpse mechanism is particularly effective in improving performance.

##### The role of object-centric representation

To examine the impact of the object-centric representation on model performance, we replaced the capsule architecture with two fully connected layers (the same size as two capsule layers; 320 and 160 units) followed by a classification readout. This new model, OCRA-no Capsules, iteratively processes the image, and its classification scores are a linear readout from the second fully connected layer representation at each timestep, which are then combined across timesteps to make the final classification decision. As shown in Table 2, accuracy for OCRA-no Capsules was lower than OCRA, highlighting the effect of object-centric representation on performance in this task. However, this effect was relatively small compared to the ablations of recurrent attention, suggesting that a recurrent model can to an extent compensate for the reduced information encapsulation that results from removal of the capsule architecture. The model has a glimpse mechanism and is free to route information globally, thereby potentially reducing the benefit derived from feature encapsulation on this task.

### Visual reasoning

**Figure 5.**
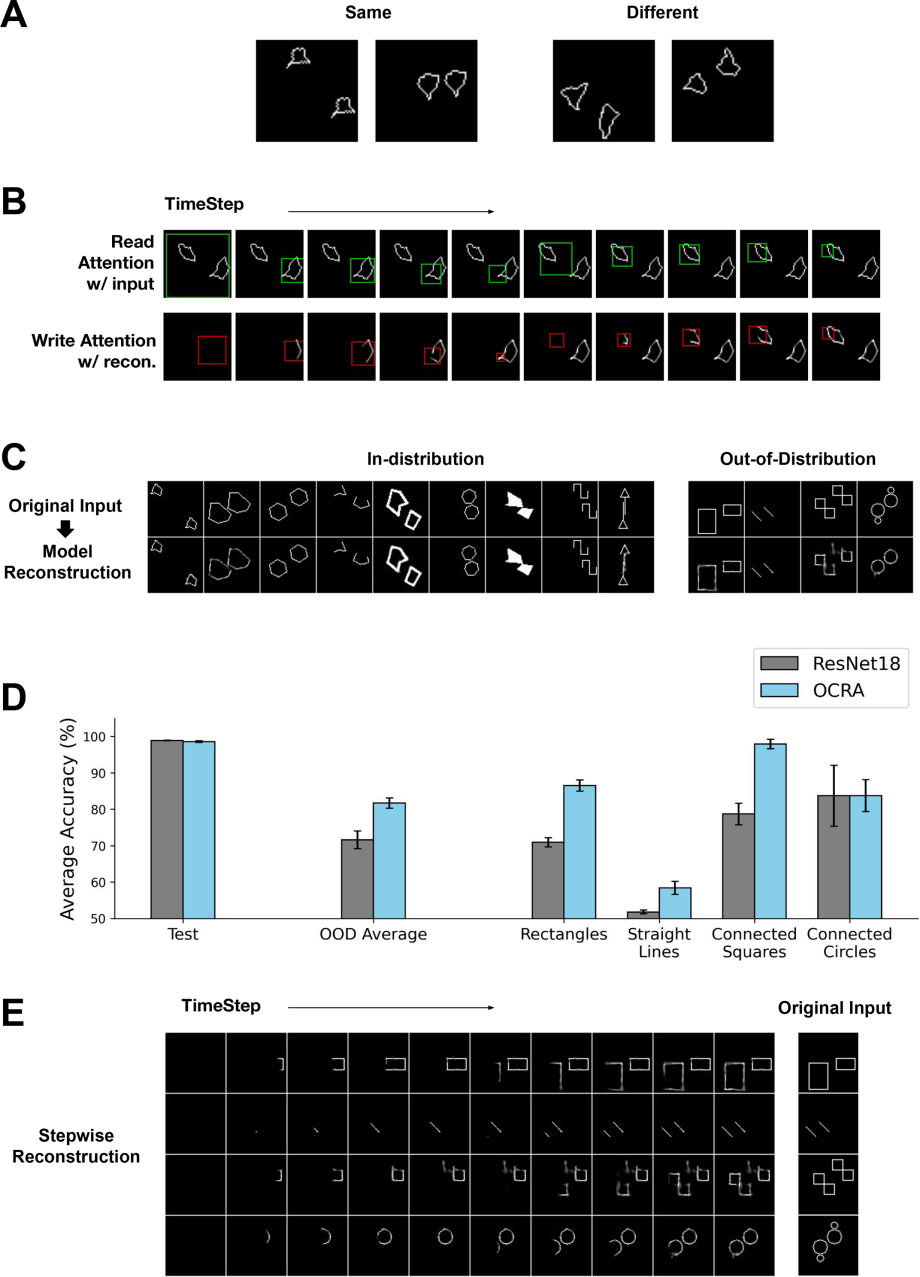
OCRA performing visual reasoning tasks. (**A**) Four sample images from SVRT Task 1, which require a same or different response. (**B**) Stepwise visualization of OCRA’s behavior on a sample test image. The top row shows the model’s read attention window in green at each time step. The bottom row shows the cumulative canvas with each write attention window in red. (**C**) Sample images and model reconstruction on the OOD generalization task with each column corresponding to a different object shape. Left: in-distribution testing dataset. Right: out-of-distribution testing dataset. (**D**) Average model accuracy for both ResNet18 (gray) and OCRA (cyan) on the original test set and the OOD test set on average, by OOD shape type. Both models achieve near-perfect accuracy on the original test set, but OCRA significantly outperforms the ResNet18 on most of the generalization tasks. Error bars show standard error. (**E**) The sequential processing of four OOD sample images, with each row corresponding to a different OOD shape. The original images are shown on the right and the stepwise cumulative reconstructions are shown on the left, which clearly illustrate object-based behavior.

The ability to reason over visual entities and the relations between them is an important cognitive ability in humans and other animals (Oden, Thompson, & Premack, 1990; Hafri & Firestone, 2021) with perhaps the ability to judge whether two objects are the same or different being the most fundamental form of visual reasoning. Fleuret et al. (2011) proposed the SVRT as a benchmark for testing this capability in artificial intelligence (AI) systems. Here our focus is on Task 1 in this benchmark (see examples in Figure 5A), which tests a model’s ability to judge whether two randomly generated objects in an image are the same or different. While behavioral results showed that humans were able to perform this task easily, early CNN models applied to this task failed to reach high levels of accuracy, thereby exposing a shortcoming (Stabinger, Rodríguez-Sánchez, & Piater, 2016; Kim, Ricci, & Serre, 2018). However, later works found that deeper CNN models (with more layers and residual connections) such as the ResNet (He, Zhang, Ren, & Sun, 2016) family of models can perform this task at near-perfect accuracy (>99%) while shallower models such as AlexNet (Krizhevsky, Sutskever, & Hinton, 2012) perform at chance level (Messina, Amato, Carrara, Gennaro, & Falchi, 2021; Funke et al., 2021). This comparison identifies computational depth as an important factor in learning this task.

Interestingly, alternative approaches have also achieved high levels of accuracy on this task despite using smaller networks. For example, siamese networks, where two networks using shared parameters are applied to the two objects in the scene, are able to learn this task (Kim et al., 2018). Placing the objects in different image channels of the input can improve the models’ performance on this task as well (Stabinger, Peer, & Rodríguez-Sánchez, 2021b). In the context of attention, these approaches are assuming a preattentive binding of features to objects by feeding the objects separately to each network (the case with siamese networks) or by providing them in separate channels. This assumption is broadly consistent with object-based attention, but it avoids the role of attention in solving the binding problem, which has long been considered a fundamental function of attention (Treisman, 1996). Consistent with OCRA and as argued by Stabinger, Peer, Piater, and Rodríguez-Sánchez (2021a) (also see Ricci, Cadène, & Serre, 2021), a more general approach would be to model the object-based attention process itself and to detect and bind the features of the objects through application of sequential processing (see Vaishnav et al., 2021, for an alternative approach for studying the role of bottom-up feature and spatial attention but focused on training efficiency on this task). Additionally, greater computational depth can be achieved through more recurrent sequential processing rather than a greater number of layers (Schwarzschild et al., 2021).

As shown in Figure 5B (top row), the model starts with a global glimpse but then immediately switches to a serial attention allocation, moving its glimpse window first to one object and then to the other. Based on this serial behavior, the first-attended object is represented by one object capsule, and this object-centric representation is maintained in the encoder–decoder RNNs across timesteps. When the model glimpses the second object, the recurrent representation would then bias the model to route information from the second object to one of two response capsule slots (same or different). This biasing is realized by a feedback connection from the decoder RNN to the encoder RNN that we added to OCRA for this task. The encoder RNN therefore uses feedforward, recurrent, and feedback inputs to update its activity as each timestep. The model is trained using the classification and reconstruction losses similar to the previous tasks. Over training, the model learns to route and reconstruct the second glimpsed object to the first response capsule if its features match those of the first object (which are being maintained through recurrent processing) and to the second response capsule if the features are different. Note that we do not have explicit working memory modules in our model, but the connection loops and the local recurrent connections do realize that function by maintaining the features of the first glimpsed object. In addition to capsules dedicated to the two objects, we also anticipated the need for a gist capsule (used most often for the initial glimpse) and perhaps a temporary object representation that forms when the glimpse moves between objects, bringing the total number of capsules used in the representational bottleneck to 4. The bottom row of Figure 5B shows the cumulative write canvas with the location and extent of the write attention for each of the 10 timesteps. Note again that OCRA does not just reconstruct the two objects serially, but its attention also gradually spreads within an object  (Jeurissen et al., 2016; Ekman et al., 2020).

Table 3 shows model accuracy on this task. OCRA achieved the same near-perfect level of accuracy (99.1%) as a ResNet18 model on this task. We then performed two ablation studies to examine the effect of the feedback connection and the object-centric representation bottleneck. The model without capsules was constructed similarly to the previous section; capsule layers were replaced by fully connected layers and a classification readout. For the no-feedback model, we simply removed the feedback connection between the decoder RNN and the encoder RNN. We found that ablating either of these components from OCRA significantly impacted its accuracy (Table 3). This shows that OCRA performs this task well by learning to leverage both an object-centric representation and a feedback mechanism for top-down biasing in order to modulate bottom-up information routing.

**Table 3.** Ablation study results on visual reasoning.

Model	Average accuracy (*SEM*)
ResNet18	99.1% (±0.43)
OCRA	99.14% (±0.38)
OCRA-no Capsules	91.38% (±2.79)
OCRA-no Feedback	92.10% (±1.81)

####  

##### Generalization to out-of-distribution stimuli

As discussed earlier, deeper CNNs can solve the same–different task in the SVRT benchmark. However, recent work revealed a major weakness (Puebla & Bowers, 2022). These authors argued that the true test of whether a model learned the concept of “sameness” is to show that it can generalize to other displays where the pixel distribution of the generated objects is different from the pixel distribution in the training set (e.g., different shapes). Models evaluated on this dataset would therefore be asked to generalize to OOD stimuli, something that humans do well. We tested OCRA and ResNet18 on a task adopted and modified from Puebla and Bowers (2022). The training set includes a collection of nine different types of stimuli, as shown in Figure 5C (left, top row). This was done to create a diverse training set to allow the models to learn a rich feature representation for performing this task. We then evaluated the models on the original test set and the OOD generalization test set, which is a collection of four types of stimuli with different shapes from the training set (examples shown in Figure 5C, right, top row).

Figure 5C (bottom row) shows examples of OCRA reconstructions for some in-distribution test samples on the left (see Supplementary Figure S2 for the cumulative canvas obtained over successive glimpses of attention for these samples) and OOD test samples on the right. As shown in Figure 5D, OCRA and the ResNet model both achieved near-perfect accuracy on the in-distribution test set for this task (leftmost bars). We found a very different result on the OOD generalization test, where OCRA significantly outperformed the ResNet model; Figure 5D shows the average accuracy over the OOD dataset and comparisons divided by the OOD test shape. These results confirm previous work (Puebla & Bowers, 2022) showing that ResNet models critically struggle with same–different judgment generalization. However, OCRA’s sequential and object-centric processing of the image generalizes to the OOD samples, as evident in the cumulative canvas for the four examples shown in Figure 5E, enabling significantly better performance. Lastly, note that OCRA’s reconstruction quality for the OOD images is not as good as for the original test images (comparing Figure 5C left and right bottom rows), showing that the model still struggles with representing these stimuli and that there is room for improvement.

## Discussion

Object-based attention endows humans with the ability to perform an unparalleled diversity of tasks, and we took inspiration from this mechanism in building OCRA. Its object-based sequential processing and recurrent computations, combined with its cognitively plausible (zoom-lens) glimpse mechanism, give OCRA processing depth comparable to larger networks. OCRA’s two-pathway architecture also took inspiration from ventral and dorsal pathways in the primate brain. Its behavior reflects an interaction between bottom-up and top-down processes such that feedforward processing is enriched and guided by current top-down hypotheses. The results show that the synthesis of these principles can lead to a model having improved robustness and generalizability, noting that we demonstrated this under challenging conditions where a mechanism for parsing the scene into entities and a recurrent process for iterative attentional sampling might prove beneficial. We also observed in the model behavior another hallmark of object-based attention, the gradual filling in of the objects (Jeurissen et al., 2016), although for now, this remains only a qualitative observation.

While many works at the intersection of machine learning and cognitive neuroscience have focused on convolutional and recurrent neural networks, encoder–decoder methods have the potential to be very useful in modeling behavior and brain responses. The generative nature of this method makes it suitable for modeling different top-down modulations and feedback processing. To date, these models have been used to study effects of top-down feedback in the ventral pathway (Svanera, Morgan, Petro, & Muckli, 2021; Al-Tahan & Mohsenzadeh, 2021) and to model predictive coding (Huang et al., 2020), mental imagery (Breedlove, St-Yves, Olman, & Naselaris, 2020), and continual learning (van de Ven, Siegelmann, & Tolias, 2020). More generally, these models can also be used for representation learning, where they can be trained using self-supervised methods to generate the visual input. In doing this, the bottleneck representation becomes a more compact representation of the image, which through the introduction of additional constraints can be disentangled and made interpretable (Storrs, Anderson, & Fleming, 2021; Higgins et al., 2021). In our work, we used this framework to model not only ventral pathway processing but also its interaction, with dorsal feedback addressing the relative paucity of work in modeling the dorsal pathway (but see Bakhtiari, Mineault, Lillicrap, Pack, & Richards, 2021, for a dual-stream architecture interpretation) compared to the ventral pathway. Our work is therefore timely and will hopefully help to close what is a gap in the cognitive modeling literature relating attention and recognition.

OCRA employed a glimpse-based (“hard”) attention mechanism as this approach has the promise of making recognition and reasoning models more accurate, efficient, and interpretable as they would only have to focus processing resources on smaller and relevant areas of the image (Mnih et al., 2014; Ba et al., 2015; Cordonnier et al., 2021; Elsayed, Kornblith, & Le, 2019; Wang, Lv, Huang, Song, Yang, & Huang, 2020; Rangrej, Srinidhi, & Clark, 2022; Papadopoulos, Korus, & Memon, 2021). An alternative approach is for the top-down processing to create spatial masks to route object-specific information from one layer to the next (Lei et al., 2021). Another approach toward modeling top-down attention has focused on “soft”-attention models that capture certain aspects of feature-based attention (Desimone & Duncan, 1995; Maunsell & Treue, 2006), where top-down modulations weight the incoming representations based on task relevance (Xu et al., 2015; Navalpakkam & Itti, 2005). In this vein, transformer-based attention mechanisms (Vaswani et al., 2017) have recently been used to create models with an integrated process of soft-attention sampling and recognition (Zoran et al., 2020; Jaegle et al., 2021), leading to better performance on adversarial images. In these models, the ongoing state representation produces queries that are matched to keys from the bottom-up processing to create attention weightings for new sampling of bottom-up values and the updating of the state representation. We believe that combining these approaches with the proposed glimpse-based mechanism helps to build a more robust model, one that not only learns an optimal policy for making attention glimpses but also biases feature processing along the processing hierarchy via top-down modulation in order to reflect current object hypotheses.

The discovery of objects in the visual input, and representing them in ways that are conducive to performing downstream tasks, has become an attractive area of research for understanding the relationship between deep learning (connectionist) approaches and human-like symbolic and object-based reasoning (Garnelo & Shanahan, 2019; Greff et al., 2020). In these models, a first stage of generating segmented representations of the objects in a scene is followed by a second stage of modeling the interaction between these objects, either through a key-query attention mechanism (similar to transformers) (Goyal et al., 2020; Ding, Hill, Santoro, Reynolds, & Botvinick, 2021) or by graph neural networks (Qi, Wang, Pathak, Ma, & Malik, 2020). These object-centric models provide the necessary representational capabilities to realize object files and make broad connections to object-based effects on behavior (Peters & Kriegeskorte, 2021; Goyal et al., 2020). These approaches allow a model to work and learn at an object-level and should be particularly useful in modeling the varied aspects of object-based attention (Pooresmaeili & Roelfsema, 2014; Vecera & O’reilly, 1998; Lei et al., 2021; George et al., 2017; Dedieu, Rikhye, Lázaro-Gredilla, & George, 2021). In return, we believe that this object-centric perspective can facilitate translation of ideas and inductive biases from the object-based attention literature, and cognitive science more generally, to build more robust and generalizable AI systems.

## Supplementary Material

Supplement 1

## Footnotes

1 github.com/hosseinadeli/OCRA and github.com/hosseinadeli/OCRA_samediff.
