# Supplementary material for: A brain-inspired object-based attention network for multiobject recognition and visual reasoning
Source: J Vis. 2023 May 22;23(5):16. doi: 10.1167/jov.23.5.16 (PMC10210512; doi:10.1167/jov.23.5.16)
Supplement: Supplement 1 [file jovi-23-5-16_s001.pdf]

A brain-inspired object-based attention network for multi-object recognition and visual reasoning

Hossein Adeli[1*], Seoyoung Ahn[1], Gregory J. Zelinsky[1,2]

[1]Department of Psychology, [2]Department of Computer Science
Stony Brook University
{hossein.adelijelodar, seoyoung.ahn, gregory.zelinsky}@stonybrook.edu
* corresponding author

1 Supplementary material

1.1 Training Regime and Hyperparameter selection

All the model training was done on a single GPU workstation. All the Models trained on the MultiMNIST task were stopped after 50 epochs of training (taking about 40 hours for the 10glimpse model). The models trained on the MultiMNIST-cluttered task were stopped after 1000 epochs (taking about 40 hours for the 5glimpse model). The models trained on the visual reasoning task were stopped after 100 epochs (taking about 2 hours).

Table 1 provides all the hyperparameters used for the three tasks. The hyperparameters were mostly the same between the tasks with a few differences. For the MultiMNIST-cluttered task, we added one background capsule and utilized a reconstruction mask, both to allow the model to focus on the main objects and ignore the background clutter. The image is larger (100×100 vs 36×36) in the MultiMNIST-cluttered task, with most of it being empty, the reconstruction loss therefore has a much smaller range compared to the other task. For this reason, and the use of the reconstruction mask, we use a much larger weight for the reconstruction loss to make it comparable to the margin loss. For this task we added a background capsule to the 10 class capsules, similar in approach to (Qin et al., 2020)). This gives the model the choice to dynamically route background noise in its attention windows to a non-class capsule, thereby allowing the model to exclude the noise from its object representations.

For the MultiMNIST task, we clip the cumulative reconstruction canvas to be between [0,1] before comparing it to the input image. This allows the model to overlap different segments in the multi-step process of writing to the canvas without increasing the loss, improving model reconstruction given the high degree of overlap between the digits.

Table 1: Hyperparameter Settings for the three tasks

Hyperparameters	MultiMNIST-(10/3)glimpse	Cluttered-(5)glimpse	Same-Different
# timesteps, t	10/3	5	10
# epoch	50	1000	100
lr	0.001	0.001	0.001
batch size	128	128	128
read glimpse size, N	18	18	18
write glimpse size, M	18	18	18
# conv1 filters	32	32	32
# conv2 filters	32	32	32
lstm size, $dim(h_{enc}), dim(h_{dec})$	512	512	512
# primary capsule	40	40	40
primary capsule dimension, $dim(p)$	8	8	8
# routings r	3	3	3
object capsule dimension, $dim(d)$	16	16	16
# background capsules	0	1	0
reconstruction loss weight, λ_{recon}	10/3	175	30
clipping final canvas to [0,1]	TRUE	FALSE	TRUE
use reconstruction mask	FALSE	TRUE	FALSE

For the Visual Reasoning task, the number of class capsules were set to 4 as explained in the main text.

1.2 Supplementary results

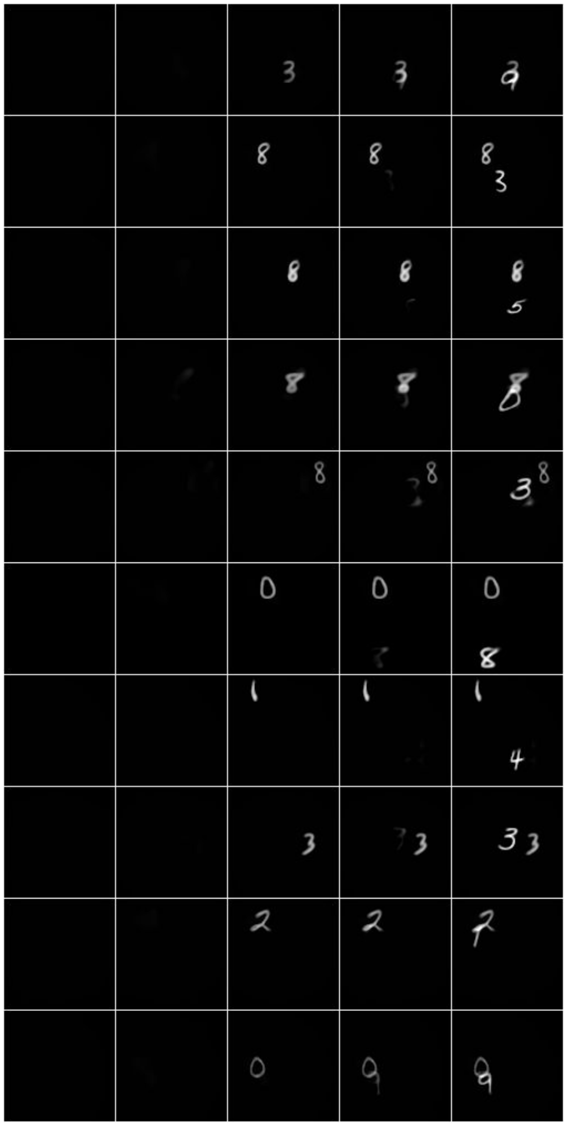

Figure 1: OCRA MultiMNIST-cluttered output with 5 glimpses showing the gradual object-based reconstruction on the cumulative canvas.

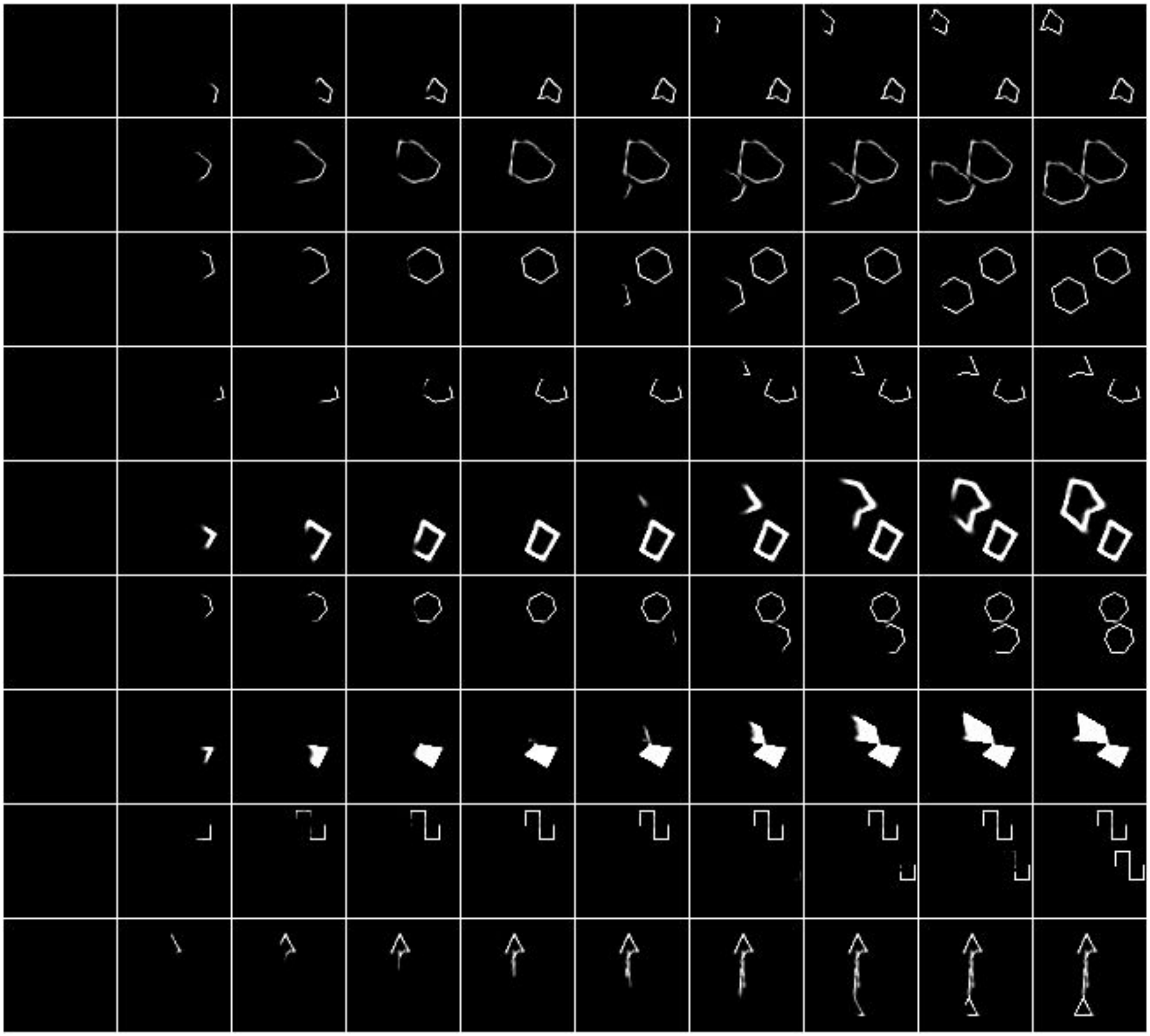

Figure 2: OCRA visual reasoning with 10 glimpses showing the gradual object-based reconstruction on the cumulative canvas.

References

Qin, Y., Frosst, N., Raffel, C., Cottrell, G., & Hinton, G. (2020). Deflecting adversarial attacks. *arXiv preprint arXiv:2002.07405*.
